# Hemolysis and Hemoglobin Structure and Function: A Team-Based Learning Exercise for a Medical School Hematology Course

**DOI:** 10.15766/mep_2374-8265.11035

**Published:** 2020-11-30

**Authors:** Arielle L. Langer, Eileen Scigliano

**Affiliations:** 1 Instructor in Medicine, Division of Hematology, Brigham and Women's Hospital; 2 Associate Professor, Department of Environmental Medicine and Public Health, Icahn School of Medicine at Mount Sinai

**Keywords:** Hematology, Hemolysis, Hemoglobin Structure, Hemoglobin Function, Physician, Pediatric Hematology-Oncology, Team-Based Learning

## Abstract

**Introduction:**

This team-based learning (TBL) exercise focused on hemolysis and hemoglobin structure and function. The goal was to emphasize content that directly impacts clinical practice, but obliges students to understand underlying pathophysiology. The readiness assurance test (RAT) covers oxygen affinity, diagnosing hemolysis, inherited causes of hemolysis (G6PD deficiency, hereditary spherocytosis, sickle cell disease, thalassemia) and acquired causes of hemolysis (thrombotic microangiopathies, autoimmune hemolytic anemia). The application activity focused on thalassemia, sickle cell disease, and autoimmune hemolytic anemia.

**Methods:**

Second-year students were divided into teams of five to six students each with one facilitator for each classroom. Students completed an individual RAT (iRAT) followed by a group RAT (gRAT). The facilitator reviewed answers of the RATs emphasizing questions where there was a lack of clarity about the correct answer. Students completed the application activity within their teams followed by a discussion guided by the facilitator.

**Results:**

On average, students answered 63% of answers correctly on the iRAT. The average team score on the gRAT was 26.7 out of 30 points. The session was well reviewed by both students and facilitators. Students ranked the quality of all facilitators as excellent with an average rating of 4.4 of 5. Exam scores improved compared to prior to the introduction of TBL, but this was also found for material not covered.

**Discussion:**

The use of TBL to emphasize the relationship between pathophysiology and the diagnosis and management of patients was both an effective teaching method and a successful way to engage medical students.

## Educational Objectives

By the end of this activity, learners will be able to:

1. Explain the clinical implications of hemoglobin structure and function.
2. Distinguish the pathophysiologic mechanisms causing hemolytic anemias.
3. Discuss the management of hemolytic anemia.

## Introduction

Team-based learning (TBL) is a teaching method focused on active participation and the application of knowledge to real world scenarios. TBL emphasizes using individual contributions to build a consensus and fosters teamwork.^1^ TBL promotes peer-to-peer teaching and exercises communication skills. These characteristics are well suited to teaching medical students, as the ultimate goal is to prepare students for patient care. In addition, active learning modalities have been shown to reduce exam failure rates,^2^ and TBL specifically has been shown to improve examination performance for students who have been struggling academically.^3^

Hemoglobin structure and function and hemolysis are central components of hematology pathophysiology covered in preclinical curricula. They represent topics in which an understanding of physiology directly informs diagnosis and management. For example, understanding the properties of Immunoglobin M compared to Immunoglobin G makes the distinct treatment of cold versus warm autoimmune hemolytic anemia intuitive. As such, this was material well suited to the applied nature of TBL, which facilitates a nuanced comparison of disorders. Hematology TBL modules are currently available for sickle cell disease and thrombotic microangiopathies topics on *MedEdPORTAL*.^4,5^ To this repertoire, we added a module covering hemolytic anemias and the clinical implications of hemoglobin structure and function rather than a specific disease. In building a module that covered this broader pathophysiologic concept, it prepared students more fully to apply concepts to clinical practice.

## Methods

### Team Formation

Assignment to sections and teams was random and shared with another preexisting TBL topic in the course. We divided the second-year class into four sections of 34–36 students, each with one facilitator. Within each section, we grouped the students into six teams of five to six students. Each section had its own classroom of six round tables, one for each team. We used this team and section size because it had been well received by students in a previous year when a different TBL topic had been added.

### Facilitator Training

We held an in-person orientation for the facilitators to ensure that they were familiar with both the pedagogical philosophy of TBL and the mechanics of making the session run smoothly. We sent the course materials to the facilitators along with suggested reading and guidelines (Appendix A) in the week prior to orientation. We held the orientation a few days prior to the TBL session to improve leader recall of details such as suggestions on how to encourage participation in a nonthreatening manner. This was also a time for facilitators to clarify learning objectives and specific teaching points of the readiness assurance test (RAT) and the application activity.

### Description of Advance Preparation Resources

Students were educated on TBL learning theory and instructed to prepare for the RATs prior to the TBL session (Appendix B). We suggested preparation with resources such as chapters 43, 46, and 47 of *Hematology: Basic Principles and Practice* by Hoffman et al^6–8^; chapters 94 and 96 of *Harrison's Principles of Internal Medicine*^9,10^; or chapters 8, 9, and 11 of *Pathophysiology of Blood Disorders* by Bunn et al.^11–13^ Preexisting syllabi or recordings of prior lectures in the hematology course at schools adopting this TBL module may also serve as a suitable resource. Students were expected to spend approximately 3 hours preparing for the session.

### Description of Readiness Assurance Process

We began the TBL session with the individual RAT (iRAT), which consisted of 10 multiple-choice questions with four answer choices on a paper handout (Appendix C). We gave students 15 minutes to complete the iRAT. The section facilitator collected the iRAT and graded it while the students completed the gRAT. This enabled facilitators to know which questions were likely to need review.

Students started the gRAT, which consisted of the same 10 questions used for the iRAT, immediately after completing the iRAT. Each team used the immediate feedback assessment technique (IF-AT) to record answers on a scratch card.^14^ Students continued to scratch off answer choices until they selected the correct answer (indicated by a star). The scratch-off cards were collected at the end of the 15 minutes allotted for the gRAT and graded after the session was completed. The gRAT score was inversely related to the number of tries required to reach the correct answer (i.e., 1 box = 3 points, 2 boxes = 2 points, 3 boxes = 1 point, 4 boxes = 0 points). After completion of the gRAT, teams were permitted to appeal the correct answer.

### Immediate Feedback and Facilitator Review of RAT

There was no immediate feedback on the iRAT. Since the gRAT was completed on an IF-AT scratch card, this provided immediate feedback for this component. After completion of the gRAT, the facilitator for each section brought the teams back together to review the 10 questions. At this point all students knew which answers were correct, thus we instructed the facilitators only to review questions for which students requested clarification. We prompted students to briefly discuss related concepts and incorrect answers, particularly if the facilitator was aware of common incorrect answers from grading the iRAT in real time. Students also received written explanations after the session was completed (Appendix D).

### Description of Team Application Activities

The application activity consisted of three detailed clinical cases with one or two multiple-choice questions following each case (Appendix E). The questions were designed to engender a discussion requiring students to use knowledge about these topics to make judgments about diagnostic or management issues. Students were given 25 minutes to work within their teams to review the cases and select answers followed by a 25-minute facilitator-led discussion. Facilitators were asked to unobtrusively listen to student discussions, make note of potential teaching points and identify teams who might be called upon to explain complicated concepts to the whole group. We adhered to the classic principles of application activities (significant problem, same problem, specific choice, simultaneous reporting)^1^ and asked the teams to all display their answer to each question simultaneously on a whiteboard during the facilitator-led review. We placed emphasis on discussing the underlying concepts for all answer choices, not just the correct answer, even if all teams chose the correct answer prior to the group discussion. Students also received written explanations of the application activity after the session was completed (Appendix F).

### Facilitation Schema

We recommended the following time allotment:

• Preparation: 3 hours.
• TBL session: 120 minutes.
  ○ iRAT: 15 minutes.
  ○ gRAT: 15 minutes.
  ○ Facilitator RAT review: 20 minutes.
  ○ Mid-point break: 5 minutes.
  ○ Application Activity: 50 minutes.
  ○ Flex time: 15 minutes.

### Evaluation of the Session

As measurements of the quality of the RAT, we used the percentage of correct answers on the iRAT and point totals on the gRAT. To assess the application activity, we received informal facilitator feedback on the quality of the discussion. Facilitator review of the RAT and application activity allowed clarification of the reasoning behind correct and incorrect answers, but also included explanation of additional concepts as directed by the students in each section. The application activity responses were not quantified.

We used performance on a section quiz and the end-of-course exam to investigate to overall impact of TBL on learning. Students took a quiz on red blood cell (RBC) disorders as well as a final exam on all the course content. The average score on questions related to the material covered in this TBL was compared to both questions on material not covered in TBL and the same questions in a prior year when TBL was not part of the course.

To evaluate the performance of the facilitators themselves, we reviewed their ratings on course evaluations. We also reviewed the students’ comments on course evaluations, which included specific questions regarding the TBL sessions.

## Results

### Session Participation and Results

With a total of 136 second-year students participating in the TBL exercise, we divided students into four sections, each section with six teams of five to six students. Two hematology/oncology fellows and two hematology/oncology faculty members served as the section facilitators. All facilitators participated in the orientation.

All students completed the iRAT. Students who qualified for additional time for testing were permitted to take the iRAT prior to the start of the session. Facilitators reported that the majority of students did not require the full 15 minutes to complete the iRAT. The average score on the iRAT was 63% (range 30%-90%). The answer distribution was displayed in Table 1. The heterogenous difficulty of questions allowed the noted spread of student performance.

**Table 1.**
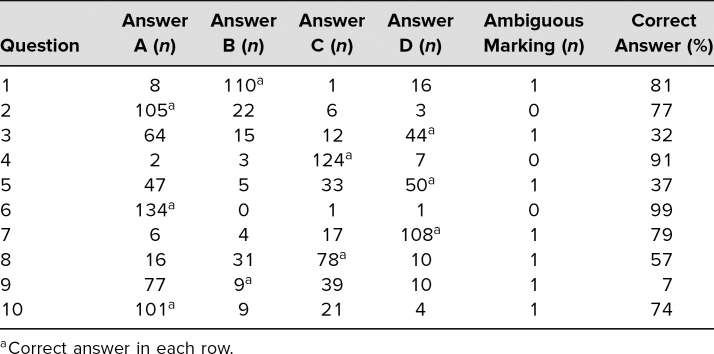
Individual Readiness Assessment Test Answer Distribution (*N* = 136)

All 24 teams completed the gRAT. Facilitators reported that the majority of teams completed the gRAT after 10 minutes, though teams were permitted the full 15 minutes allotted. The average score on the gRAT was 26.7 out of 30 points (89%; range of 23–29 points), better than the iRAT performance, as is typically seen in TBL exercises. The distribution of attempts to achieve a correct answer on the gRAT was displayed in Table 4. Two of the 10 questions frequently required multiple attempts by the teams.

### Exam Scores

On the RBC disorders quiz, students got 85% of questions related to TBL material correct as compared with 87% of questions not related to TBL material. This 85% was an improvement from 75% in a prior year; however, the non-TBL performance had also improved from a prior 75%. Similarly, on the final exam the average score on items corresponding to this TBL was 87% compared to 92% for other items. This was a modest improvement compared to a prior year, when the average was 84%, while the average score from the non-TBL material was previously 91%.

### Student Evaluations and Facilitator Feedback

As part of the course feedback and evaluation, students gave the facilitator's ability to facilitate learning a score of 4.4 of 5 and their rapport with students 4.4 of 5. The range of scores was narrow for both items, 4.3–4.5 and 4.3–4.6, respectively. These ratings were also comparable to the average ratings for faculty leading traditional small groups used elsewhere in the course but with less heterogeneity, with 4.4 of 5 (range 3.6–4.9) for facilitating learning and 4.4 of 5 (range 3.4–4.9).

A recurring theme in feedback from students was that the facilitator review of the RAT questions should be briefer and focus primarily on questions that students struggled with during the team discussions. Students reported that time spent on this portion of TBL felt redundant and unnecessary. However, facilitators reported these discussions sometimes revealed misunderstandings regarding the reasoning behind correct answers and were helpful to ensure that all students had achieved the desired depth of understanding.

Students reported a positive association with this TBL including difficult questions with comments such as, “It gave me a lot of opportunities to test my understanding of the material,” and “Questions tested our knowledge and made sure that we were keeping up with the course material.” They also expressed an appreciation of the peer teaching component saying, “Extremely helpful to work in small groups with my classmates to reason things through,” and “One of us will likely know the information and it is much less intimidating than talking through questions with an MD.”

## Discussion

We developed a TBL exercise on hemolysis and hemoglobin structure and function that was well regarded by both students and facilitators. This exercise can be used to introduce a first TBL session or expand the TBL in a medical school hematology curriculum.

As we created the session, we focused on the application to clinical practice, which motivated students to be engaged with the material. We found that a brief facilitator orientation was helpful in ensuring that both the logistics of and the discussion during the session were optimized. This orientation may also account for the high and narrow range of ratings received by the facilitators as compared to ratings of traditional small-group leaders. Student comments helped identify that an emphasis on avoiding redundancy when reviewing the RATs was a vital component of facilitator training, though this needs to be balanced with diligence that all students achieve an adequate level of comprehension.

We identified several limitations of our evaluation. Most importantly, we did not see a clear improvement on exam scores neither during the RBC disorders quiz nor the final exam relative to other items or to historical performance on these questions. Given the variably difficulty of questions on the exam historically and the fact that the topic chosen for TBL was not more complex than some other RBC disorders, we were unable to ascertain whether this reflected the exam questions themselves or the quality of the TBL. Another limitation was that one item on the RAT (question 6) was answered correctly by a very high proportion of students (Table 1), which indicated it was likely too easy to foster learning. Conversely, question 9 was answered correctly by only a small proportion of students and this improved only modestly on the gRAT (Tables 1 and 2), indicating it may be more difficult than ideal. There was the option for teams to appeal answers; however, no teams chose to appeal answers during the facilitator review. Additionally, while we believe student ratings highlighted the importance and impact of facilitator orientation, the uniformity of ratings could simply reflect the structure of TBL dominating impressions. Furthermore, while we perceived these ratings as positive, these were subjective assessments, and it remains possible that facilitators were insufficiently trained to run the sessions in an optimal manner. Finally, while we received feedback from the facilitators on common incorrect answers during the application activity, we failed to quantify these answers to augment our explanations (Appendix F).

**Table 2.**
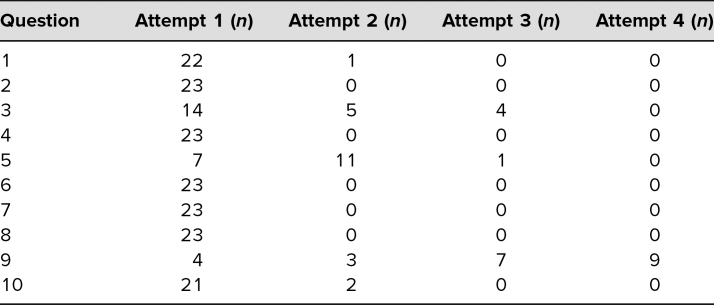
Group Readiness Assessment Test Distribution of Attempts to Achieve Correct Answer (*N* = 23)

The learning of hemolysis and hemoglobin structure and function are well suited to TBL, as understanding the physiology is directly applicable to diagnosis and management. By covering a sufficiently broad topic rather than a single disease, this session was more analogous to clinical practice and more feasible to include in preclinical curricula that usually have only a few weeks to teach all of hematology. Incorporating this session into medical school curricula is likely to be of benefit to students.

## Appendices

Facilitator Guide.docxStudent Guide.docxiRAT gRAT Questions.docxiRAT gRAT Answers.docxApplication Activity Questions.docxApplication Activity Explanations.docx
All appendices are peer reviewed as integral parts of the Original Publication.
