# Supplementary material for: Hemolysis and Hemoglobin Structure and Function: A Team-Based Learning Exercise for a Medical School Hematology Course
Source: MedEdPORTAL. 2020 Nov 30;16:11035. doi: 10.15766/mep_2374-8265.11035 (PMC7703478; doi:10.15766/mep_2374-8265.11035)
Supplement: Supplementary file 1 — Facilitator Guide.docxStudent Guide.docxiRAT gRAT Questions.docxiRAT gRAT Answers.docxApplication Activity Questions.docxApplication Activity Explanations.docx [file mep_2374-8265.11035-s001.zip › A. Facilitator Guide.docx]

**Team Based Learning Facilitator Guide**

**Basic Concept:**

Team based learning is an interactive small group exercise that promotes application of knowledge, clinical reasoning, peer to peer teaching and communication skills.

**Time and Location:**

INSERT TIME AND LOCATION Hemolytic Anemias and Hemoglobin Disorders

**Grading:**

Attendance is mandatory. Each session is worth INSERT THE WEIGHT IN YOUR COURSE.

Grading will be based on a combination of scores from Individual and Group Readiness Assurance Tests.

**Facilitator Preparation**

Recommended reading:

Michaelsen L, Sweet M. The essential elements of team-based learning. *New Directions of Teaching and Learning*. 2008;2008(116):7-27.^1^

Facilitator orientation: INSERT TIME AND LOCATION

Written explanations for the iRAT/gRAT questions will be provided to instructors.

Teaching Points and explanations for the application activity will be provided to instructors.

**Format:** Students are divided into sections of 30-36 students and then within sections into groups of 5-6 students. Each sections will have one facilitator.

**Part 1: Individual (pre-session) preparation**

Student preparation (review of relevant materials posted on blackboard) is required prior to attendance at the TBL session. Students are allotted 3 hours of course time to prepare (denoted as **TBL Prep time** on course calendar) for each TBL session. Of course, students may use additional time to review materials.

Students are asked to review:

INSERT INSTRUCTIONS FOR ACCESSING YOUR COURSE SYLLABUS AND ANY OTHER RESOURCE YOU HAVE AVAILABLE (e.g. prior year slides, prior recorded lectures from your institution) OR ONE OF THESE REFERENCE MATERIALS:

- Hematology: Basic Principles and Practice by Hoffman et al, Chapters 43, 46 & 47^2-4^
- Harrison’s Principles of Internal Medicine, Chapters 94 & 96^5,6^
- Pathophysiology of Blood Disorders by Bunn et al, Chapters 8, 9 & 11^7-9^

**Part 2 Individual readiness assurance test (iRAT) (closed book): 15 minutes**

The iRAT portion of the TBL exercise consists of 10 multiple choice questions

Each student will **work individually** to complete the iRAT portion of the TBL module.

Students have 15 minutes to complete and submit the written question sheet. This is closed book.

During the gRAT portion of the exercise, instructors are asked to grade the iRATs and indicate the score on the top of the student question sheet.

If all students are done prior to 15 minutes, move on to the gRAT.

**Part 3 Group readiness assurance test (gRAT) (closed book): 15 minutes**

The gRAT portion of the TBL exercise consists of the **same 10 multiple** **choice questions used in the iRAT.** Students will **work together** with their team members to answer these questions. This is closed book.

Using the provided **scratch-off answer cards,** as a team, students will decide which answer to select.

When the selected choice is scratched off there will be a star if the answer is correct.

If the answer is incorrect, students will then make an additional selection.

Students continue to select choices until they reach the correct answer.

The score is based on the number of attempts needed to pick the correct answer.

1 attempt 🡪 3 points

2 attempts 🡪 2 points

3 attempts 🡪1 point

4 attempts 🡪0 points

As a team, students have 15 minutes to complete and submit the gRAT portion of the exercise.

Students should total their gRAT score and record on the scratch off card.

**Team Number should be written on the top of the scratch off card.**

If all groups are done prior to 15 minutes, move on to the facilitator feedback.

**Part 4: Facilitator Feedback: 20 minutes**

The facilitator will briefly review the multiple choice questions with all the teams together.

Not all questions need to be reviewed. If all teams answered a question correctly and the content seems to be clear, you do not need to review the question. Take a pause for each question to provide an opportunity to ask questions, but move onto the next question without discussion if the students don’t have any questions. The students will be provided written explanations of iRAT/gRAT after the session.

**Part 5: Application Activity (open book): 25 minutes**

The application activity is a series of multiple choice questions intended to elicit discussion prompting students to apply knowledge they have acquired (from prior preparation and from discussions during the gRAT and faculty feedback parts of the TBL session).

The application activity classically follows the 4 S’s:^1^

Activity addresses a **S**ignificant problem

Students are all working on the **S**ame problem

Students make a **S**pecific choice

Students all report their answer to each question **S**imultaneously

Hard copies of the questions are provided to each team.

Students work with their teams to arrive at an answer for each question. While the groups are discussing the questions, the facilitator should walk around the room to eavesdrop on the discussions and identify common areas of confusion or teams that may be able to articulate difficult concepts for the full section discussions.

**Part 6: Application Activity Review (open book): 25 minutes**

The groups will come back together to discuss the questions as a full section. The facilitator will ask the teams to simultaneously report their answers to each question (wipe boards are provided.) The instructor should record the tally for each answer choice on the white board, so that the distribution of answers is apparent to all groups.

Time is taken after each question to have teams explain how they arrived at their answers even if all teams answer correctly to be certain that everyone understands the thought process. Students should also be prompted to explain why incorrect answers are wrong, if this helps engrain key concepts or allows refinement of the understanding of correct answer.

Written explanations of the application activity questions will be provided to the students after the session.

**References**

1. Michaelsen L, Sweet M. The essential elements of team-based learning. *New Directions of Teaching and Learning.* 2008;2008(116):7-27.
2. Benz EJ, Ebert BL. Hemoglobin variants associated with hemolytic anemia, altered oxygen affinity, and methemoglobinemias. In: Hoffman R, Benz EJ, Silberstein LE, et al eds. *Hematology: Basic Principles and Practice*. 7th ed. Elsevier; 2018:608-615. <https://doi.org/10.1016/C2013-0-23355-9>
3. Michel M, Jäger U. Autoimmune hemolytic anemia. In: Hoffman R, Benz EJ, Silberstein LE, et al eds. *Hematology: Basic Principles and Practice*. 7th ed. Elsevier; 201:648-662.el. <https://doi.org/10.1016/C2013-0-23355-9>
4. Mentzer WC, Schrier SL. Extrinsic nonimmune hemolytic anemias. In: Hoffman R, Benz EJ, Silberstein LE, et al eds. *Hematology: Basic Principles and Practice*. 7th ed. Elsevier; 2018:663-672. <https://doi.org/10.1016/C2013-0-23355-9>
5. Benz EJ. Disorders of hemoglobin. In: Jameson JL, Fauci AS, Kasper DL, Hauser SL, Longo DL, Loscalzo J, eds. *Harrison's Principles of Internal Medicine*. 20th ed. McGraw-Hill Education; 2018.
6. Luzzatto L. Hemolytic anemias. In: Jameson JL, Fauci AS, Kasper DL, Hauser SL, Longo DL, Loscalzo J, eds. *Harrison's Principles of Internal Medicine*. 20th ed. McGraw-Hill Education; 2018.
7. Bunn HF, Sankaran VG. Thalassemia. In: Aster JC, Bunn HF, eds. *Pathophysiology of Blood Disorders*. 2nd ed. McGraw-Hill Education; 2016:93-108.
8. Bunn HF. Acquired hemolytic anemias. In: Aster JC, Bunn HF, eds. *Pathophysiology of Blood Disorders*. 2nd ed. McGraw-Hill Education; 2016:137-146.
9. Bunn HF. Sickle cell disease. In: Aster JC, Bunn HF, eds. *Pathophysiology of Blood Disorders*. 2nd ed. McGraw-Hill Education; 2016:109-124.
