# Supplementary material for: Hemolysis and Hemoglobin Structure and Function: A Team-Based Learning Exercise for a Medical School Hematology Course
Source: MedEdPORTAL. 2020 Nov 30;16:11035. doi: 10.15766/mep_2374-8265.11035 (PMC7703478; doi:10.15766/mep_2374-8265.11035)
Supplement: Supplementary file 1 — Facilitator Guide.docxStudent Guide.docxiRAT gRAT Questions.docxiRAT gRAT Answers.docxApplication Activity Questions.docxApplication Activity Explanations.docx [file mep_2374-8265.11035-s001.zip › B. Student Guide.docx]

**Team Based Learning Student Guide and Learning Objectives**

**Introduction**

Team based learning (TBL) is an interactive exercise that promotes application of knowledge, clinical reasoning, peer to peer teaching and communication skills.

A review of the theory and practice of TBL, Michaelsen and Sweet’s *The Essential Elements of Team-Based Learning New Directions for Teaching and Learning,* is posted with the TBL materials on INSERT COURSE WEBSITE

**Time and Location**

INSERT TIME

Sessions will take place in INSERT LOCATION

Students are divided into teams of 5-6 students each. There are 5-6 teams in each room.

Please **see TBL roster** (CREATE ROSTER) for assigned team and room.

One student from each team has been randomly assigned to be the team leader.

**Grading**

Attendance is mandatory. Each session is worth INSERT VALUATION IN COURSE.

Grading will be based on scores from Individual and Group Readiness Assurance Tests.

**Format**

**Part 1: Individual (pre-session) preparation**

Student preparation (review of relevant materials) is required prior to attendance at the TBL session.

INSERT LINK TO COURSE SYLLABUS, PRIOR LECTURES OR ONE OF THESE REFERENCE MATERIALS:

- - Pathophysiology of Blood Disorders by Bunn et al, Chapters 8, 9 & 11
  - Hematology: Basic Principles and Practice by Hoffman et al, Chapters 43, 46 & 47
  - Harrison’s Principles of Internal Medicine, Chapters 94 & 96

**Part 2 Individual readiness assurance test (iRAT) (15 minutes)**

The iRAT portion of the TBL exercise consists of 10 multiple choice questions.

**This is a closed book exercise.**

Each student will **work individually** to complete the iRAT portion of the TBL module.

Students have 15 minutes to complete and submit the written question/answer sheet.

**Part 3 Group readiness assurance test (gRAT) (15 minutes)**

The gRAT portion of the TBL exercise consists of the **same 10 multiple** **choice questions used in the iRAT.** Students will **work together** with their team members to answer these questions.

**This is a closed book exercise .**

Using the provided **scratch-off answer cards,** as a team, students will decide which answer to select.

When the selected choice is scratched off there will be a star if the answer is correct.

If the answer is incorrect, students will then make an additional selection.

Students continue to select choices until they reach the correct answer.

The score is based on the number of attempts needed to pick the correct answer.

1 attempt 🡪 3 points

2 attempts 🡪 2 points

3 attempts 🡪1 point

4 attempts 🡪0 points

As a team, students have 10 minutes to complete and submit the gRAT portion of the exercise.

**Record the total gRAT score on the top of the card.**

**Please make sure to record your TEAM Number on the gRAT card before submitting.**

**Part 4: Facilitator Feedback (20 minutes)**

The facilitator will **briefly** **review only those questions** that were **difficult or confusing** for most students. **Students may appeal incorrect answers** with arguments defending their choice.

Key learning points for each question will be summarized.

**Part 5: Application activity (50 minutes: 25 minutes to select answers, 25 minutes for review)**

The application activity is a series of multiple choice questions (using clinical scenarios) intended to prompt students to apply knowledge they have acquired (from prior preparation and discussions during the gRAT and faculty feedback parts of the TBL session).

Hard copies of the questions are provided to each team.

Students work with their teams to arrive at an answer for each question.

At the end of the time limit, the instructor will ask the teams to simultaneously report their answers to each question (wipe boards are provided.)

Time is taken after each question to have teams explain how they arrived at their answers.

**Written explanations for the iRAT and application activity questions will be posted on INSERT COURSE WEBSITE after the session is completed.**

**Learning Objectives**

**Hemolytic Anemias**

1. Describe the RBC life cycle and the steps of hemoglobin degradation

2. Explain the general pathophysiologic mechanisms of **intra**vascular and **extra**vascular hemolysis Describe the biochemical consequences and clinical manifestations that result from each.

3. Describe the pathophysiologic mechanisms underlying specific hemolytic disorders including:

Immune mediated hemolysis (warm vs cold)

Microangiopathic hemolysis (Disseminated intravascular coagulation, thrombotic thrombocytopenic purpura, HELLP syndrome)

Red blood cell membrane disorders (hereditary spherocytosis, paroxysmal nocturnal hemoglobinuria, spur cell anemia))

Red blood cell enzyme disorders (G6PD deficiency, pyruvate deficiency)

4. Describe the clinical setting, manifestations and diagnostic testing that help distinguish among the different hemolytic disorders.

5. Identify the following RBC abnormalities on a blood smear and explain what causes the abnormal RBC shape:

Spherocyte

Schistocyte

Acanthocyte (spur cell)

Bite cells

Target cells

Sickle cells

**Hemoglobin Disorders**

**Sickle Cell Disease**

1. Explain the pathogenesis of sickle cell disease: genetic mutation, properties of hemoglobin S, role of endothelium and nitric oxide.

2. Explain factors that lead to increased sickling of red blood cells

3. Describe the clinical manifestations (organ, by organ) of sickle cell anemia (homozygous state) and how each of these can be related to the properties of Hgb S.

4. Distinguish sickle cell anemia from sickle cell trait.

5. Distinguish sickle cell anemia from sickle-beta thalassemia and hemoglobin C disease.

6. Describe the findings on hemoglobin electrophoresis for:

Sickle cell anemia (SS) in a patient who has never been transfused

Sickle cell anemia (SS) in a patient who is getting red blood cell transfusions

Sickle cell trait (AS)

Sickle-beta thalassemia

(What would be the approximate proportions of Hgb A, Hgb A_2_, Hgb F and Hgb S)

7. Discuss treatments for sickle cell anemia.

8. Identify sickled cells, target cells, nucleated red blood cells, Howell Jolly bodies on a peripheral blood smear. Explain why they occur.

**Thalassemias (alpha and beta)**

1. Explain the pathogenesis of the thalassemia syndromes

2. Explain the biochemical and clinical consequences of unbalanced globin chain synthesis.

3. Explain the pathophysiologic mechanism underlying the clinical manifestations of thalassemias:

Hemolytic anemia

Microcytic, hypochromic anemia

Hepatosplenomegaly

Skeletal abnormalities (marrow expansion)

Iron Overload

4. Describe the typical findings on hemoglobin electrophoresis for:

Beta thalassemia (major and minor)

Sickle-beta thalassemia

Alpha thalassemia trait

Hgb H disease (alpha thalassemia with 3 gene defect)

5. Discuss treatment of beta thalassemia major (Cooley’s anemia)

6. Discuss the clinical consequences of chronic transfusion therapy.
