# Supplementary material for: Hemolysis and Hemoglobin Structure and Function: A Team-Based Learning Exercise for a Medical School Hematology Course
Source: MedEdPORTAL. 2020 Nov 30;16:11035. doi: 10.15766/mep_2374-8265.11035 (PMC7703478; doi:10.15766/mep_2374-8265.11035)
Supplement: Supplementary file 1 — Facilitator Guide.docxStudent Guide.docxiRAT gRAT Questions.docxiRAT gRAT Answers.docxApplication Activity Questions.docxApplication Activity Explanations.docx [file mep_2374-8265.11035-s001.zip › C. iRAT gRAT Questions.docx]

**Hemolytic Anemias and Hemoglobin Disorders TBL: iRAT/gRAT Questions**

**Student Name______________________________________ iRAT Score________**

1. A patient develops sepsis and respiratory failure manifested by fever, hypotension, bacteremia and hypoxemia. Which of the following explains the ability of hemoglobin (Hgb) to improve delivery of oxygen to the tissues in this clinical situation?
   1. An increase in temperature shifts the Hgb-oxygen dissociation curve to the left.
   2. An increase in 2, 3-DPG decreases Hgb’s affinity for oxygen.
   3. Hypoxemia leads to increased levels of Hgb F which has a decreased affinity for oxygen.
   4. Acidosis leads to oxidation of the heme iron molecule enabling it to reversibly bind oxygen.
2. Which of the following distinguishes intravascular from extravascular hemolysis?
   1. Hemoglobinuria
   2. Decreased haptoglobin
   3. Elevated LDH
   4. Elevated indirect bilirubin
3. A 32 year old woman in the 3^rd^ trimester of her first pregnancy presents with severe vaginal bleeding. Due to a clerical error she is given type A-positive blood even though her blood type is B-positive. Which of the following is a potential complication of this clinical scenario?
   1. The patient will develop extravascular hemolysis
   2. The fetus is likely to develop hemolysis
   3. The patient may develop liver failure
   4. The patient may develop renal failure
4. Which hemoglobin electrophoresis pattern is consistent with sickle cell trait?

|  | % Hgb A | % Hgb A2 | % Hgb S |
| --- | --- | --- | --- |
| a. | 0 | 2 | 98 |
| b. | 98 | 2 | 0 |
| c. | 60 | 0 | 40 |
| d. | 30 | 0 | 70 |

1. A new patient in your office reports being diagnosed with thalassemia major as a child, but doesn’t recall which type. Which finding on hemoglobin electrophoresis would help you distinguish alpha thalassemia major from beta thalassemia major?
   1. Elevated hemoglobin H
   2. Elevated hemoglobin F
   3. Elevated hemoglobin A2
   4. All of the above
2. Which of the following induces sickling in patients with sickle cell anemia?
   1. Hypoxemia
   2. Increased Hemoglobin F
   3. Folic acid
   4. Transfusion
3. A 25 year old woman is referred for evaluation of anemia. Laboratory tests reveal:

WBC 5,000/ul Hgb 10.5 gm/dL Hct 29.5% MCV 65 fl Platelets 250,000/ul. Serum iron, total iron binding capacity and ferritin levels are normal. A CBC from a year ago is the same.

Which of the following is the most likely diagnosis?

a. Anemia of Inflammation

b. Iron deficiency anemia

c. Pernicious anemia

d. Thalassemia minor

1. A 27 year old man is evaluated in the office for sudden onset dark urine. Four days ago the patient began taking trimethoprim-sulfamethoxazole for bacterial sinusitis. He has a brother who developed hemolysis when exposed to a sulfa-containing drug. On physical exam he is noted to be tachycardic and icteric. There is no hepatosplenomegaly.

Which of the following is also found in this disease?

a. The presence of spherocytes on the blood smear

b. Positive Coombs test

c. Heinz bodies on special staining

d. Autosomal dominant inheritance pattern

1. A previously healthy 46 year old woman presents with shortness of breath, petechiae on her legs and nose bleeding. A CBC shows anemia and thrombocytopenia. Serum chemistries show normal hepatic function. Review of the blood smear shows schistocytes. What other abnormality is likely to be present in this patient?
   1. Prolongation of clotting times (PT and PTT)
   2. Neurologic symptoms
   3. Splenomegaly
   4. Bloody diarrhea
2. A 28 year old man is referred to a hematologist for evaluation of a chronic, ‘life-long’ anemia. He had a cholecystectomy for gallstones 3 years ago, but otherwise has been well. Physical examination is remarkable for mild jaundice and a palpable spleen tip. Review of blood smear shows polychromasia and spherocytes. Which of the following is the mechanism most likely responsible for this patient’s anemia?

- 1. Mutation leading to reduced red blood cell membrane elasticity
  2. Mutation limiting hemoglobin synthesis
  3. Antibody binding to red blood cells leading to enhanced splenic macrophage removal
  4. Loss of red blood cell decay accelerating factor leading to enhanced complement lysis of red blood cells
