# Supplementary material for: Hemolysis and Hemoglobin Structure and Function: A Team-Based Learning Exercise for a Medical School Hematology Course
Source: MedEdPORTAL. 2020 Nov 30;16:11035. doi: 10.15766/mep_2374-8265.11035 (PMC7703478; doi:10.15766/mep_2374-8265.11035)
Supplement: Supplementary file 1 — Facilitator Guide.docxStudent Guide.docxiRAT gRAT Questions.docxiRAT gRAT Answers.docxApplication Activity Questions.docxApplication Activity Explanations.docx [file mep_2374-8265.11035-s001.zip › D. iRAT gRAT Answers.docx]

**Hemolysis and Hemoglobin Disorders iRAT/gRAT Answers and Explanations**

1. A patient develops sepsis and respiratory failure manifested by fever, hypotension, bacteremia and hypoxemia. Which of the following explains the ability of hemoglobin (Hgb) to improve delivery of oxygen to the tissues in this clinical situation?
   1. An increase in temperature shifts the Hgb-oxygen dissociation curve to the left.
   2. An increase in 2, 3-DPG decreases Hgb’s affinity for oxygen.*
   3. Hypoxemia leads to increased levels of Hgb F which has a decreased affinity for oxygen.
   4. Acidosis leads to oxidation of the heme iron molecule enabling it to reversibly bind oxygen.

**Answer:** **B.** Increased 2,3 DPG decreases Hemoglobin’s affinity for oxygen (Hgb-oxygen dissociation curve shifts to the right) favoring unloading of oxygen to the tissues. Similarly, increased temperature (fever) shifts the curve to the right (NOT to the left, answer a), decreasing Hgb’s oxygen affinity and favoring oxygen unloading. Hgb F has an INCREASED affinity for oxygen (answer c) which makes it ideal to be the primary form of hemoglobin in utero where there is a need for fetal hemoglobin to be able to grab up oxygen from the maternal circulation in the placenta. In order for the heme iron to reversibly bind oxygen it must be in the reduced, NOT oxidized, state (answer d.)

Answer D was the most common incorrect answer with 11 percent of students choosing this option on the iRAT.

1. Which of the following distinguishes intravascular from extravascular hemolysis?
   1. Hemoglobinuria*
   2. Decreased haptoglobin
   3. Elevated LDH
   4. Elevated indirect bilirubin

**Answer:** **A**. Answers b, c and d are all present in either type of hemolysis. Hemoglobinuria is only seen in intravascular hemolysis where hemoglobin is released directly into the circulation (hemoglobinemia) and is then cleared, in part, by the kidney resulting in hemoglobinuria. During clinically significant extravascular hemolysis, enough hemoglobin leaks into the circulation to deplete haptoglobin (answer b).

Answer B was the most common incorrect answer with 16 percent of students choosing this option on the iRAT.

1. A 32 year old woman in the 3^rd^ trimester of her first pregnancy presents with severe vaginal bleeding. Due to a clerical error she is given type A-positive blood even though her blood type is B-positive. Which of the following is a potential complication of this clinical scenario?
   1. The patient will develop extravascular hemolysis
   2. The fetus is likely to develop hemolysis
   3. The patient may develop liver failure
   4. The patient may develop renal failure*

**Answer:** **D.** The scenario describes an ABO major mismatch blood transfusion which can result in acute intravascular hemolysis. Anti-A IgM antibodies in the recipient’s plasma (she is blood type B and therefore has anti-A antibodies) will bind to donor A RBCs, fix complement (leading to complement activation) and cause the RBCs to lyse immediately, within the circulation i.e. intravascular hemolysis (not extravascular, answer a.). IgM antibodies (unlike IgG) are too large to cross the placenta and the fetus will not be affected (answer b). Acute renal failure may result from hemoglobinuria: Hgb is released directly into the circulation and is cleared by kidney. Heme proteins are thought to precipitate cast formation and obstruction of renal tubules. Liver failure does not occur (answer c.)

Answer A was the most common incorrect answer with 47 percent of students choosing this option on the iRAT, which is more than the 32 percent who chose the correct option, answer D. Thirty-nine percent (9/23) of teams answered incorrectly (at least 2 attempts needed) on the gRAT.

1. Which hemoglobin electrophoresis pattern is consistent with sickle cell trait?

|  | % Hgb A | % Hgb A2 | % Hgb S |
| --- | --- | --- | --- |
| a. | 0 | 2 | 98 |
| b. | 98 | 2 | 0 |
| c.* | 60 | 0 | 40 |
| d. | 30 | 0 | 70 |

**Answer: C**. Choice A is a patient with Hgb SS. Choice B is a normal electrophoresis pattern. Sickle cell trait patients always have less than 50% Hgb S because of shorter survival of red cells enriched for Hgb S compared to Hgb A. In addition, alpha globin chains preferentially bind to normal beta globin chains (vs sickle beta globin chains) resulting in the disproportionate amount of Hgb A (vs Hgb S) in the trait setting. Choice D likely represents a patient with Hgb SS who has received a blood transfusion (lowering the level of Hgb S). Untransfused patients with Hgb SS usually have >95% Hgb S with the remainder of their hemoglobin comprised of Hgb A2 and/or Hgb F and cannot make their own Hgb A.

Answer D was the most common incorrect answer with 5 percent of students choosing this option on the iRAT.

1. A new patient in your office reports being diagnosed with thalassemia major as a child, but doesn’t recall which type. Which finding on hemoglobin electrophoresis would help you distinguish alpha thalassemia major from beta thalassemia major?
   1. Elevated hemoglobin H
   2. Elevated hemoglobin F
   3. Elevated hemoglobin A2
   4. All of the above*

**Answer: D**. Hemoglobin H (tetramer of 4 beta chains) would only be present in alpha thalassemia major (or intermedia). Elevation of Hgb F and Hgb A2 can both be seen in beta thalassemia major, but would not be seen in alpha thalassemia. In the beta thalassemias there is a compensatory increase in non-beta containing hemoglobins (F and A2). This would not be possible in alpha thalassemias since alpha globin chain production is impaired.

Answer A was the most common incorrect answer with 35 percent of students choosing this option on the iRAT, while incorrect answer C was select by 24 percent of students. The correct choice, answer D was still the most common at 37 percent. Fifty-two percent (12/23) of teams answered incorrectly (at least 2 attempts needed) on the gRAT.

1. Which of the following induces sickling in patients with sickle cell anemia?
   1. Hypoxemia*
   2. Increased Hemoglobin F
   3. Folic acid
   4. Transfusion

**Answer:** **A.** Since only deoxygenated hemoglobin sickles, hypoxemia can increase sickling. Increased amounts of Hgb F within the RBC decreases the concentration of Hgb S which decreases sickling. Some therapeutic strategies in SCA seek to increase Hgb F levels (e.g. Hydroxyurea). Transfusion increases the proportion of RBCs that contain normal Hgb A (not Hgb S) and therefore decrease risk of sickling. Folic acid may be deficient in patients with chronic hemolytic anemias and thus these patients should all be on folate supplementation. But, folate does not promote sickling.

Answers C and D were each selected by only one student each (<1 percent each) on the iRAT with 99 percent of students selecting the correct choice, answer A.

1. A 25 year old woman is referred for evaluation of anemia. Laboratory tests reveal:

WBC 5,000/ul Hgb 10.5 gm/dL Hct 29.5% MCV 65 fl Platelets 250,000/ul. Serum iron, total iron binding capacity and ferritin levels are normal. A CBC from a year ago is the same.

Which of the following is the most likely diagnosis?

a. Anemia of Inflammation

b. Iron deficiency anemia

c. Pernicious anemia

d. Thalassemia minor*

**Answer:** **D**. Microcytic anemia with normal iron studies is classic for thalassemia. The level of the patient’s hemoglobin indicates thalassemia minor, not major. The stability of her hemoglobin over the past year is also suggestive of thalassemia. Serum iron would be expected to be low for choice a or b. Ferritin would be expected to be low for choice b and high for choice a. The MCV should be high, not low, for pernicious anemia, which leads to B12 deficiency.

Answer C was the most common incorrect answer with 13 percent of students choosing this option on the iRAT.

1. A 27 year old man is evaluated in the office for sudden onset dark urine. Four days ago the patient began taking trimethoprim-sulfamethoxazole for bacterial sinusitis. He has a brother who developed hemolysis when exposed to a sulfa-containing drug. On physical exam he is noted to be tachycardic and icteric. There is no hepatosplenomegaly.

Which of the following is also found in this disease?

a. The presence of spherocytes on the blood smear

b. Positive Coombs test

c. Heinz bodies on special staining*

d. Autosomal dominant inheritance pattern

**Answer:** **C.** This patient appears to have hemolysis following exposure to a sulfa drug, which is classic for **G6PD deficiency**. A male relative with the same condition is further support for this inherited disorder, which is **X-linked** recessive (not autosomal dominant-answer d). Patients with G6PD have **Heinz bodies**, which represent **hemoglobin denatured by oxidative stress, precipitated within the RBC** (and on the RBC membrane). It is uncommon for autoimmune hemolytic anemia (choice b) to run in families and is not associated with TMP-SMX or bacterial sinusitis. TMP-SMX is not associated with drug-induced autoimmune hemolysis. The presence of spherocytes (answer a) suggests hereditary spherocytosis, a condition in which hemolysis is not usually triggered by medications or bacterial sinusitis, is usually chronic not acute, and often, though not always, causes splenomegaly (work hypertrophy from chronic extravascular hemolysis). Spherocytes should be distinguished from the microspherocytes seen in warm autoimmune hemolytic anemia (i.e. direct coombs positive for IgG) by their size, though as already delineated for the direct coombs, microspherocytes would also not be expected.

Answer B was the most common incorrect answer with 23 percent of students choosing this option on the iRAT.

1. A previously healthy 46 year old woman presents with shortness of breath, petechiae on her legs and nose bleeding. A CBC shows anemia and thrombocytopenia. Serum chemistries show normal hepatic function. Review of the blood smear shows schistocytes. What other abnormality is likely to be present in this patient?
   1. Prolongation of clotting times (PT and PTT)
   2. Neurologic symptoms*
   3. Splenomegaly
   4. Bloody diarrhea

**Answer: B.** The most likely diagnosis in a previously healthy patient with new onset of a microangiopathic hemolytic anemia (MAHA), thrombocytopenia with normal hepatic function, and no evidence of sepsis is **thrombotic thrombocytopenic purpura (TTP).** TTP was originally described by a Mount Sinai physician, Dr. Eli Moschcowitz, in 1925. **A pentad of: microangiopathic hemolysis, thrombocytopenia, renal insufficiency, fever and neurologic symptoms** were the classic clinical findings. MAHA with prolongation of clotting times (answer a.) would be characteristic of disseminated intravascular coagulation. DIC is ALWAYS secondary to an underlying, usually catastrophic, event associated with tissue injury such as overwhelming infection, obstetrical complications (abrupted/ruptured placenta), surgery. The patient presents without any indication of a preceding severe illness or event. In TTP, vessels are obstructed by platelet (not fibrin) “clots”. Clotting factors are not consumed and therefore clotting times (PT, PTT) are normal. MAHA associated with bloody diarrhea is characteristic of hemolytic uremic syndrome (HUS) associated with shiga-toxin (E.coli) infection typically seen in children.

Answer A was the most common incorrect answer with 57 percent of students choosing this option on the iRAT, while incorrect answer C was select by 29 percent of students. The correct choice, answer D was an uncommon selection at 7 percent in the iRAT but improved to 17 percent (4/23) of teams selecting the correct answer on the first attempt and 13 percent selecting the correct answer on the second attempt on the gRAT.

1. A 28 year old man is referred to a hematologist for evaluation of a chronic, ‘life-long’ anemia. He had a cholecystectomy for gallstones 3 years ago, but otherwise has been well. Physical examination is remarkable for mild jaundice and a palpable spleen tip. Review of blood smear shows polychromasia and spherocytes. Which of the following is the mechanism most likely responsible for this patient’s anemia?

- 1. Mutation leading to reduced red blood cell membrane elasticity*
  2. Mutation limiting hemoglobin synthesis
  3. Antibody binding to red blood cells leading to enhanced splenic macrophage removal
  4. Loss of red blood cell decay accelerating factor leading to enhanced complement lysis of red blood cells

**Answer:** **A.** The most likely diagnosis **is chronic extravascular hemolysis from hereditary spherocytosis**. The chronic, life-long anemia suggests an inherited condition. The presence of jaundice, splenomegaly (work hypertrophy from chronic extravascular hemolysis) and cholecystectomy (bilirubin gallstones from chronic hyperbilirubinemia) supports this diagnosis. The presence of spherocytes on the smear suggests the etiology. **Hereditary spherocytosis is caused by a variety of mutations that affect membrane proteins such as ankyrin, spectrin and band 3 that impact RBC membrane elasticity**. These mutations lead to loss of deformability and premature destruction of the RBCs. These mutations do not affect hemoglobin synthesis (answer b). Spherocytes may result from autoimmune hemolytic anemia (answer c), but this is an acquired condition, typically with an acute onset, and, in about 50% of cases, associated with an underlying cause (e.g. drug-induced, lymphoma-associated, collagen vascular disease-associated). Loss of decay accelerating factor (answer d) is the defect in paroxysmal nocturnal hemoglobinuria), an acquired disorder leading to episodic intravascular hemolysis. Splenomegaly and spherocytosis are not features of PNH.

Answer C was the most common incorrect answer with 15 percent of students choosing this option on the iRAT.
