# Supplementary material for: Hemolysis and Hemoglobin Structure and Function: A Team-Based Learning Exercise for a Medical School Hematology Course
Source: MedEdPORTAL. 2020 Nov 30;16:11035. doi: 10.15766/mep_2374-8265.11035 (PMC7703478; doi:10.15766/mep_2374-8265.11035)
Supplement: Supplementary file 1 — Facilitator Guide.docxStudent Guide.docxiRAT gRAT Questions.docxiRAT gRAT Answers.docxApplication Activity Questions.docxApplication Activity Explanations.docx [file mep_2374-8265.11035-s001.zip › E. Application Activity Questions.docx]

**Hemolytic Anemias and Hemoglobin Disorders TBL Application Activity**

**Case # 1**

A 6 year old girl presents to your clinic with increasing abdominal girth, yellowing of the eyes and

fatigue. Her mother reports that multiple other family members have similar symptoms. She has never

had any episodes of painful crises and has never required a blood transfusion. Her exam is notable for

conjunctival pallor, mild scleral icterus and splenomegaly. Her laboratory evaluation reveals:

WBC Count 5.2 x 10^3^/uL

Hemoglobin 9.9 g/dL

Hematocrit 30 %

RBC Count 5.0 x10^6^/uL

MCV 60 fL

MCH 20 pg

RDW 13.8 %

Platelets 358 x 10^3^/uL

Retic Count 9 %

LDH 400 u/L

Total Bilirubin 3.5 mg/dL

Direct Bilirubin 0.4 mg/dL

AST 33 u/L

ALT 23 u/L

Fe 70 mcg/dL

TIBC 100 mcg/dL

Transferrin Sat 70 %

Ferritin 215 ng/mL

Direct Coombs Negative

Review of her blood smear reveals pale, small, uniform-appearing RBCs, polychromasia and target cells

Which of the following should be included in the management of the underlying condition?

1. Folic acid supplementation
2. Prevention of painful crises with hydroxyurea
3. Chelation for iron overload
4. Surveillance for pigment gallstones
5. Corticosteroids
6. All of the above
7. None of the above
8. 1 and 5 only
9. 1, 2 and 4 only
10. 1, 3 and 4 only

**Case # 2 Question 1**

A 23 year old man with sickle cell anemia presents to the ED with several days of abdominal, chest, lower back and left hip pain. He has a fever, pleuritic chest pain and a productive cough. He has been prescribed hydroxyurea and folate supplementation. He was last seen by his hematologist 6 months ago. He recently returned to New York after visiting his sister in Peru where they spent time hiking in the Andes. He has no other medical problems. On physical exam the patient is ill appearing, alert, oriented, mildly tachypneic with some crackles on lung exam. No JVD, adenopathy, peripheral edema, calf pain or swelling. He is admitted to the hospital for evaluation. His labs on admission to the ED and those from his visit to his hematologist 6 months ago are shown.

|  | Last Hematology Visit | Emergency Department |
| --- | --- | --- |
| WBC Count (x 10^3^/uL) | 10 | 12 |
| Hemoglobin (g/dL) | 8.8 | 6.5 |
| Hct (%) | 32 | 20 |
| RBC count (x 10^6^/ul) | 3 | 2 |
| Mean Corpuscular Volume (fl) | 106 | 100 |
| Platelets (x 10^3^/uL) | 300 | 300 |
| Reticulocyte Count (%) | 10 | 4 |
| Total Bilirubin (mg/dL) | 2.5 | 5.8 |
| Direct Bilirubin (mg/dL) | 0.2 | 0.8 |
| LDH (u/L) | 300 | 450 |

Which of following factors may be contributing to the patient’s current clinical picture (current symptoms and worsening anemia)?

- - 1. Low atmospheric oxygen tension
    2. Bohr effect
    3. Splenic sequestration
    4. Iron deficiency
    5. Medication non-adherence

1. 2, 3, 5
2. 1, 3, 4
3. 1, 2, 5
4. 2, 3, 4
5. 1, 4, 5

**Case # 2 Question #2**

The patient is started on hydration, pain medication and antibiotics, but continues to have pleuritic chest pain. He develops SOB, tachycardia, and worsening hypoxemia. Vital signs reveal: T 101.2^o^ F, P 102, BP 106/68, RR 20. A chest x ray reveals a new infiltrate. You obtain blood cultures and a repeat CBC, which shows that his hemoglobin in now 4.9 g/dL.

What is the next best step in managing this patient?

- 1. Ventilation/Perfusion (V/Q) scan and heparin
  2. Simple transfusion
  3. Corticosteroids
  4. Exchange transfusion

**Case # 3 There are 2 clinical scenarios (Patient 1 and Patient 2)**

**Patient 1**

A 48 year old woman presents to the Emergency Department with a several week history of progressive shortness of breath. In the past few days she also noticed her eyes yellowing and a rash on her cheeks. She has no known past medical history. On review of systems, she reports intermittent joint pain for the past year. Her vital signs are normal. Her exam is notable for scleral icterus, conjunctival pallor, a malar rash, and a spleen palpable ~3cm below the left costal margin.

WBC Count 5.6 x 10^3^/uL

Hemoglobin 8.1 g/dL

Hematocrit 24%

RBC Count 2.5 x10^6^/uL

MCV 96 fL/cell

Platelet count 183 x 10^3^/uL

Reticulocyte Count 8%

LDH 601 u/L

Total Bilirubin 4.1 mg/dL

Direct Bilirubin 0.4 mg/dL

AST 33 u/L

ALT 38 u/L

Direct Coombs Positive for IgG (negative for C3)

Haptoglobin Decreased

**Patient 2**

A 61 year old man with diabetes and hypertension initially presented to his PCP 10 days ago with fever and cough. He was diagnosed with pneumonia and started on Azithromycin. His fevers have resolved, but he feels short of breath and fatigued. A repeat CXR shows improvement in the previously noted bilateral patchy opacities. His exam is notable for scleral icterus, conjunctival pallor, and scattered rhonchi in bilateral lung fields. His spleen is not palpable. Laboratory studies show:

WBC count 9.2 x 10^3^/uL

Hemoglobin 9.5 g/dL

Hematocrit 28 %

RBC Count 3.0 x10^6^/uL

MCV 93 fL

Platelet count 314 x 10^3^/uL

Reticulocyte Count 11%

LDH 511 u/L

Total Bilirubin 3.9 mg/dL

Direct Bilirubin 0.2 mg/dL

AST 42 u/L

ALT 37 u/L

Direct Coombs Positive for C3 (negative for IgG)

Haptoglobin Decreased

Which of the following are appropriate therapeutic options for these two patients?

1. Folic acid
2. Corticosteroids
3. Splenectomy after failure of other therapies
4. Transfusion if anemia becomes more severe
5. All of the above are appropriate for both patients
6. All of the above are appropriate for Patient 1, but only A is appropriate for Patient 2
7. All of the above are appropriate for Patient 2, but only A is appropriate for Patient 1
8. All of the above are appropriate for Patient 1, but only A and D are appropriate for Patient 2
9. All of the above are appropriate for Patient 2, but only A and D are appropriate for Patient 1
