# Supplementary material for: Hemolysis and Hemoglobin Structure and Function: A Team-Based Learning Exercise for a Medical School Hematology Course
Source: MedEdPORTAL. 2020 Nov 30;16:11035. doi: 10.15766/mep_2374-8265.11035 (PMC7703478; doi:10.15766/mep_2374-8265.11035)
Supplement: Supplementary file 1 — Facilitator Guide.docxStudent Guide.docxiRAT gRAT Questions.docxiRAT gRAT Answers.docxApplication Activity Questions.docxApplication Activity Explanations.docx [file mep_2374-8265.11035-s001.zip › F. Application Activity Explanations.docx]

**Hemolytic Anemias and Hemoglobin Disorders Explanations TBL Application Activity**

**Case # 1**

A 6 year old girl presents to your clinic with increasing abdominal girth, yellowing of the eyes and

fatigue. Her mother reports that multiple other family members have similar symptoms. She has never

had any episodes of painful crises and has never required a blood transfusion. Her exam is notable for

conjunctival pallor, mild scleral icterus and splenomegaly. Her laboratory evaluation reveals:

WBC Count 5.2 x 10^3^/uL

Hemoglobin 9.9 g/dL

Hematocrit 30 %

RBC Count 5.0 x10^6^/uL

MCV 60 fL

MCH 20 pg

RDW 13.8 %

Platelets 358 x 10^3^/uL

Retic Count 9 %

LDH 400 u/L

Total Bilirubin 3.5 mg/dL

Direct Bilirubin 0.4 mg/dL

AST 33 u/L

ALT 23 u/L

Fe 70 mcg/dL

TIBC 100 mcg/dL

Transferrin Sat 70 %

Ferritin 215 ng/mL

Direct Coombs Negative

Review of her blood smear reveals pale, small, uniform-appearing RBCs, polychromasia and target cells

Which of the following should be included in the management of the underlying condition?

1. Folic acid supplementation
2. Prevention of painful crises with hydroxyurea
3. Chelation for iron overload
4. Surveillance for pigment gallstones
5. Corticosteroids
6. All of the above
7. None of the above
8. 1 and 5 only
9. 1, 2 and 4 only
10. 1, 3 and 4 only*

**Teaching points - Learning Objectives:**

1. Identify patient with thalassemia
2. Distinguish thalassemia from other microcytic anemias (disorders of impaired hemoglobin synthesis such as iron deficiency and anemia of inflammation) and other inherited hemolytic anemias (e.g. sickle cell anemia).
3. Identify iron overload and explain risk in thalassemia (even in absence of transfusion)
4. Identify the need for iron chelation in thalassemia
5. Explain the importance of folate supplementation in diseases that cause increased RBC turnover
6. Explain the link between chronic hemolysis and risk of pigment gallstone formation

**Explanation: Answer E is correct.** This patient has thalassemia. Thalassemia is an inherited disorder resulting in a decreased synthesis of (qualitatively normal) globin chains. Unbalanced globin chain synthesis (e.g. patients with beta thalassemia don’t make enough beta globin resulting in an excess of alpha globin chains) contributes to the clinical consequences of thalassemias.

- **Why thalassemia?**
  - The presence of anemia, indirect bilirubinemia, elevated LDH, reticulocytosis (absolute retics 450,000/ul; retic index 3.7) suggest **hemolysis.**
  - Signs and symptoms in a young child with a similar condition in other family members makes an **inherited disorder** likely.
  - The presence of **splenomegaly** indicates that hemolysis is chronic and extravascular (work hypertrophy). Extramedullary hematopoiesis is a constant feature of thalassemia that also contributes to splenomegaly.
  - The **low MCV, low MCH and normal RDW** are consistent with the **blood smear findings** of a pale (**hypochromic**), small (**microcytic**), **uniform** (normal red cell distribution width) population of RBCs. Microcytosis, hypochromia and target cells are features of anemias caused by **impaired hemoglobin synthesis**: iron deficiency, thalassemias, anemia of inflammation. Target cell formation occurs when there is an increase in the amount of RBC membrane relative to intracellular contents. In disorders of impaired hemoglobin synthesis, that ratio is increased because of decreased intracellular hemoglobin.
  - The iron studies show **mild iron overload** which is a common feature of thalassemia intermedia and thalassemia major. **Thalassemias are associated with increased gut uptake of iron due to low hepcidin levels.** In addition, **chronic hemolytic anemias lead to iron accumulation** (**ongoing breakdown of hemoglobin releases iron)** since the body has no normal mechanism of excreting excess iron.
- **Why not iron deficiency?**
  - The elevated reticulocyte count, iron studies showing mild iron overload (rather than deficiency) and the normal RDW are **inconsistent** with iron deficiency. The RDW is typically elevated in iron deficiency reflecting the decreasing size of RBCs over time as iron deficiency develops.
- **Why not anemia of inflammation?**
  - Anemia of inflammation is secondary (in part) to unavailable iron leading to impaired Hgb production and is typically characterized by reticulocytopenia and low serum iron levels. The patient does not have an underlying chronic inflammatory disorder to explain AI.
- **Why not sickle cell anemia?**
  - The absence of painful crises, sickled cells on blood smear and the presence of microcytosis and hypochromia are inconsistent with SCA. SCA is not a disorder of impaired (quantitative) synthesis of hemoglobin and RBCs are not hypochromic and microcytic. Target cells are often seen in SCA secondary to intracellular dehydration leading to an increase in ratio of membrane to intracellular contents.
- **Why not autoimmune hemolytic anemia?**
  - The negative Coombs test is inconsistent with AIHA. Although spherocytes are present in the blood in AIHA, they are usually not so numerous as to cause the MCV to be so low. AIHA is not a “microcytic” anemia.

We can infer that the diagnosis is most likely **thalassemia intermedia** based on the clinical presentation: age, degree of anemia, lack of need for transfusion (?yet). The clinical presentation of alpha thalassemia intermedia (Hemoglobin H disease) and beta thalassemia intermedia may be quite similar. Hemoglobin electrophoresis and genetic testing will be diagnostic.

**Folate supplementation:** in patients with **active hemolysis,** **demand for folic acid is increased** (needed for DNA synthesis and cell turnover) to support the bone marrow’s compensatory increase in RBC production. Folate supplementation is vital to avoid folate deficiency which would compromise bone marrow production of cells

**Iron chelation** is a common adjunctive therapy for thalassemia. One might debate whether she needs to start chelation at the first visit, but a child who already has evidence of iron overload at age 6 will definitely need it soon to prevent end organ damage.

**Pigment gallstones:** Due to **increased production of bilirubin** any patient with **chronic hemolysis** has **an** increased risk of pigment gallstones. It is important to remember this risk factor because pigment stones are harder to pick up on imaging than traditional calcium-containing stones and can be missed if this part of the history isn’t considered.

**Corticosteroids** would be an appropriate treatment for warm (IgG) autoimmune hemolytic anemia, but has no role in treatment of thalassemia.

**Hydroxyurea** is a treatment for sickle cell disease.

**Case # 2 Question 1**

A 23 year old man with sickle cell anemia presents to the ED with several days of abdominal, chest, lower back and left hip pain. He has a fever, pleuritic chest pain and a productive cough. He has been prescribed hydroxyurea and folate supplementation. He was last seen by his hematologist 6 months ago. He recently returned to New York after visiting his sister in Peru where they spent time hiking in the Andes. He has no other medical problems. On physical exam the patient is ill appearing, alert, oriented, mildly tachypneic with some crackles on lung exam. No JVD, adenopathy, peripheral edema, calf pain or swelling. He is admitted to the hospital for evaluation. His labs on admission to the ED and those from his visit to his hematologist 6 months ago are shown.

|  | Last Hematology Visit | Emergency Department |
| --- | --- | --- |
| WBC Count (x 10^3^/uL) | 10 | 12 |
| Hemoglobin (g/dL) | 8.8 | 6.5 |
| Hct (%) | 32 | 20 |
| RBC count (x 10^6^/ul) | 3 | 2 |
| Mean Corpuscular Volume (fl) | 106 | 100 |
| Platelets (x 10^3^/uL) | 300 | 300 |
| Reticulocyte Count (%) | 10 | 4 |
| Total Bilirubin (mg/dL) | 2.5 | 5.8 |
| Direct Bilirubin (mg/dL) | 0.2 | 0.8 |
| LDH (u/L) | 300 | 450 |

Which of following factors may be contributing to the patient’s current clinical picture (current symptoms and worsening anemia)?

- - 1. Low atmospheric oxygen tension
    2. Bohr effect
    3. Splenic sequestration
    4. Iron deficiency
    5. Medication non-adherence

1. 2, 3, 5
2. 1, 3, 4
3. 1, 2, 5*
4. 2, 3, 4
5. 1, 4, 5

**Teaching points - Learning Objectives:**

1. Identify pathophysiologic causes of sickling
2. Explain metabolic consequences of chronic hemolytic anemias (vis a vis iron, folate).
3. Explain pathophysiologic (anatomic) consequences of vaso-occlusive crises.

**Explanation: Answer C is correct.** This patient with sickle cell anemia (SS disease) presents with signs and symptoms of a painful vaso-occlusive crisis, pneumonia (fever, pleuritic chest pain, cough) and worsening anemia following a recent trip to Peru where he went hiking in the Andes.

**What contributed to:**

- **Vaso-occlusive crisis:** obstruction of small blood vessels leading to tissue infarction and pain
  - **Low atmospheric oxygen tension** (e.g. travel to high altitude such as Peru, long plane flights) will decrease the proportion of hemoglobin that is bound to oxygen. **Increased deoxyhemoglobin enhances polymerization of Hgb S** and increases sickling and risk for VOC.
  - **Infection** or **focal ischemia** leading to **acidemia or local acidosis** in the tissues diminishes the affinity of hemoglobin for oxygen (the **Bohr effect**). Focal ischemia also triggers the local production of 2,3 DPG, which also decreases Hgb affinity for oxygen. Both **acidosis and 2,3DPG** shift the Hgb-Oxygen dissociation curve to right, a **decreased affinity for and unloading of oxygen from Hgb**. Again, increase in deoxyhemoglobin enhances polymerization of Hgb S, sickling and VOC.
  - **Increased temperature (fever)** similarly leads to a shift of the Hgb-Oxygen dissociation curve to the right with unloading of oxygen, increase in deoxyHgb and more sickling.
  - **Hemolysis** contributes to the vasculopathy of SCA since free heme (from Hgb breakdown) scavenges nitric oxide leading to vasoconstriction and endothelial damage (increased adherence of sickled RBCs to endothelium 🡪 VOC).
  - **Hydroxyurea** decreases the incidence of VOC and is FDA approved for use in patients with SCA**. If the patient stopped taking HU** (*as* ***suggested* by decreasing MCV**) this may have contributed to VOC. HU inhibits ribonucleotide reductase leading to impaired DNA synthesis (megaloblastosis and macrocytosis**). Patients taking HU will have an elevated MCV** and this finding is reliable enough to be routinely used as a **measure of adherence**. The lower MCV currently (vs 6 months ago) suggests that perhaps he has stopped taking HU.
  - **Worsening anemia (see below):** Anything that worsens anemia will worsen tissue oxygen delivery, which leads to a higher rate of oxygen extraction; that means a greater proportion of hemoglobin will be deoxygenated in the post capillary venules. We know that since only deoxygenated sickle hemoglobin can sickle, this increases the risk of a sickle cell crisis
- **Worsening anemia**
  - **Reticulocytopenia** (absolute retics 80,000/ul, down from 300,000/ul 6 months earlier) suggests suppressed bone marrow function. **Folate deficiency** (leading to impaired erythropoiesis) is **common in patients with chronic hemolytic anemias** because folate demands are higher. Patients don’t always realize the importance of folate supplementation, so **medication non-adherence** is usually the cause when such patients become folate deficient.
  - **Infection** can suppress bone marrow function. **Parvovirus** (not an answer choice) infects erythroid precursors (in the marrow) leading to **red cell aplasia and severe reticulocytopenia** and worsening of anemia. This can be particularly problematic in patients with chronic hemolytic anemias who depend on active marrow erythropoiesis to maintain Hgb levels.
  - **Active hemolysis,** as indicated by the higher indirect bilirubin and elevated LDH, may lead to worsening of anemia. More active sickling (see causes above) leads to more hemolysis.
  - **Iron deficiency is unlikely.** Patients with **chronic hemolysis are at risk for iron overload**. When Hgb is broken down following hemolysis of the RBC, the released iron is recycled to make more Hgb or stored in macrophages (in liver). The body has **no normal mechanism for excreting excess iron.** The only way to lose iron from the body is through bleeding. When stores are full, iron starts to accumulate in the parenchyma of organs: liver, heart, endocrine glands. This is hemochromatosis.
  - **Splenic sequestration will not occur in a** 26 year old with SCA (Hgb SS disease) who has an infarcted, shrunken spleen. Splenic sequestration crisis (massive intrasplenic sickling with trapping of large blood volume within the spleen) is a life-threatening complication of SCA that only occurs in infants and children with functioning spleens.

**(NB: patients with SC disease** (double heterozygotes for Hgb S and Hgb C) tend to have less active sickling (than SS patients) and **continue to have splenomegaly** into adulthood, so they **may develop splenic sequestration**)

**Case # 2 Question #2**

The patient is started on hydration, pain medication and antibiotics, but continues to have pleuritic chest pain. He develops SOB, tachycardia, and worsening hypoxemia. Vital signs reveal: T 101.2^o^ F, P 102, BP 106/68, RR 20. A chest x ray reveals a new infiltrate. You obtain blood cultures and a repeat CBC, which shows that his hemoglobin in now 4.9 g/dL.

What is the next best step in managing this patient?

- 1. Ventilation/Perfusion (V/Q) scan and heparin
  2. Simple transfusion*
  3. Corticosteroids
  4. Exchange transfusion

**Teaching points - Learning Objectives:**

- 1. Recognize acute chest syndrome
  2. Distinguish acute chest syndrome from pulmonary embolism
  3. Explain the risks and benefits of simple versus exchange transfusion in patients with sickle cell anemia

**Explanation: Answer B is correct.** The patient has worsening respiratory symptoms, progressive hypoxemia and worsening anemia in the presence of a pulmonary infiltrate. The main differential diagnosis includes acute chest syndrome (ACS) and pulmonary embolism (PE).

**Which is the next best step in managing this patient?**

- **V/Q scan and heparin:** if a PE is strongly suspected, confirmation with diagnostic testing would be indicated and, if confirmed, initiation of anticoagulation would be appropriate. The presence of an **infiltrate on CXR makes PE a less likely** diagnosis (vs ACS). While the presence of calf pain and/or swelling is sensitive (though not specific) for deep vein thrombosis, they may be absent in patients with venous thrombo-embolism (VTE). The **patient has no edema/pain/swelling, however, his recent plane flight is a potential risk factor for VTE** (stasis). In the presence of an infiltrate, a CT angiogram, rather than V/Q scan would be a better diagnostic test for PE. In this patient, the pretest probability (**Wells et al Thrombosis and Haemostasis 2000**) for PE is low. A V/Q scan and heparin would not be the best next step.
- **Simple transfusion vs Exchange transfusion:** Acute chest syndrome is a life-threatening complication in patients with SCA resulting from vaso-occlusion within the pulmonary microvasculature. ACS may be difficult to distinguish with certainty from pneumonia. In a patient with **worsening respiratory symptoms, hypoxemia, pulmonary infiltrate** with active (or recent) vaso-occlusive crisis **despite antibiotics**, **ACS must be strongly considered** and treated. The **primary treatment for ACS** (in addition to hydration, pain management, supplemental oxygen) is to **decrease the level of Hemoglobin S to stop sickling**. **In patients who are very anemic, this can be accomplished with simple blood transfusions**. For patients with higher hemoglobin levels**, increasing the hemoglobin above 10 g/dL (with transfusion) may exacerbate the viscosity of the blood and compound the risk of sickling**. In such a situation, **exchange transfusion, which involves first removing some blood from the patient before giving a blood transfusion**, is indicated. Exchange transfusion requires a special nurse and a central venous catheter (risk of infection and pneumothorax), and more units of RBCs (6-10 units). In this patient, **simple transfusion would be the treatment of choice given the severe anemia (Hgb 4.9 g/dL).**
- **Corticosteroids** do not have a role in the management of acute chest syndrome or pulmonary embolism.

**Case # 3 There are 2 clinical scenarios (Patient 1 and Patient 2)**

**Patient 1**

A 48 year old woman presents to the Emergency Department with a several week history of progressive shortness of breath. In the past few days she also noticed her eyes yellowing and a rash on her cheeks. She has no known past medical history. On review of systems, she reports intermittent joint pain for the past year. Her vital signs are normal. Her exam is notable for scleral icterus, conjunctival pallor, a malar rash, and a spleen palpable ~3cm below the left costal margin.

WBC Count 5.6 x 10^3^/uL

Hemoglobin 8.1 g/dL

Hematocrit 24%

RBC Count 2.5 x10^6^/uL

MCV 96 fL/cell

Platelet count 183 x 10^3^/uL

Reticulocyte Count 8%

LDH 601 u/L

Total Bilirubin 4.1 mg/dL

Direct Bilirubin 0.4 mg/dL

AST 33 u/L

ALT 38 u/L

Direct Coombs Positive for IgG (negative for C3)

Haptoglobin Decreased

**Patient 2**

A 61 year old man with diabetes and hypertension initially presented to his PCP 10 days ago with fever and cough. He was diagnosed with pneumonia and started on Azithromycin. His fevers have resolved, but he feels short of breath and fatigued. A repeat CXR shows improvement in the previously noted bilateral patchy opacities. His exam is notable for scleral icterus, conjunctival pallor, and scattered rhonchi in bilateral lung fields. His spleen is not palpable. Laboratory studies show:

WBC count 9.2 x 10^3^/uL

Hemoglobin 9.5 g/dL

Hematocrit 28 %

RBC Count 3.0 x10^6^/uL

MCV 93 fL

Platelet count 314 x 10^3^/uL

Reticulocyte Count 11%

LDH 511 u/L

Total Bilirubin 3.9 mg/dL

Direct Bilirubin 0.2 mg/dL

AST 42 u/L

ALT 37 u/L

Direct Coombs Positive for C3 (negative for IgG)

Haptoglobin Decreased

Which of the following are appropriate therapeutic options for these two patients?

1. Folic acid
2. Corticosteroids
3. Splenectomy after failure of other therapies
4. Transfusion if anemia becomes more severe
5. All of the above are appropriate for both patients
6. All of the above are appropriate for Patient 1, but only A is appropriate for Patient 2
7. All of the above are appropriate for Patient 2, but only A is appropriate for Patient 1
8. All of the above are appropriate for Patient 1, but only A and D are appropriate for Patient 2*
9. All of the above are appropriate for Patient 2, but only A and D are appropriate for Patient 1

**Teaching points – Learning Objectives:**

1. Identify the clinical and laboratory features of autoimmune hemolytic anemia
2. Distinguish between “warm” and “cold” mediated autoimmune hemolytic anemia.
3. Explain how the pathophysiology of the hemolysis determines the appropriate therapeutic intervention.

**Explanation: Answer D is correct.** Both patients have evidence of hemolysis: increased indirect bilirubin, increased LDH, decreased haptoglobin and reticulocytosis. The underlying cause (and pathophysiology) of the hemolysis will determine the appropriate therapy.

- **The mechanism for hemolysis is immune-mediated in both patients:**
  - **In patient 1,** the **positive direct Coombs test for IgG** indicates that IgG **antibodies are bound to the patient’s RBCs**. The clinical history of **malar rash and joint pains** suggests a collagen vascular disease such as **systemic lupus erythematosus** (SLE) which may be associated with **autoimmune hemolytic anemia.** This type of AIHA is considered **“warm”** because the **IgG antibodies have a greater affinity** (thermal amplitude) **to bind to RBCs** **in the warmer parts of the body** (core vs extremities). The **hemolysis is extravascular. IgG coated RBCs are removed from the circulation** (in pieces, giving rise to spherocytes, or completely) **when bound to Fc receptors on macrophages within the red pulp of the spleen (i.e. outside the circulation).**  The presence of s**plenomegaly** supports this mechanism as the spleen has enlarged through **work hypertrophy**. IgG is not as good at binding and activating complement (leading to intravascular hemolysis) as IgM. So**, typically IgG immune mediated hemolysis is extravascular** (not intravascular through complement activation).
  - **In patient 2,** the **direct Coombs is positive for C3** (complement). The patient presented 10 days earlier with **community acquired pneumonia** (CAP) and now has evidence of hemolysis. One of the most common causes of CAP is **mycoplasma pneumoniae.** Mycoplasma infection is **often associated with autoimmune hemolysis. Hemolysis is mediated by IgM antibodies** against the “I” (big “I”) antigen on RBCs. This IgM antibody is called a **cold agglutinin** as it binds to RBCs best in colder areas of the body (extremities). **IgM actively binds complement** (**positive direct Coombs for C3**) **sometimes leading to complement activation**. Because IgM falls off the RBC in warmer temps as the Ab-bound RBCs circulate away from the colder extremities back to the warmer body core, it is not detected by the Coombs test. Hemolysis occurs **both extravascularly** when **macrophages phagocytose the complement-coated RBCs** (macrophages have complement receptors) **and** through **activation of the complement cascade leading to intravascular hemolysis**. The macrophages tend to engulf the entire complement-coated RBC and **thus spherocytes are not a feature of cold hemolysis** (I don’t know why). **Splenomegaly is not a feature of cold IgM mediated-hemolysis because** this type of hemolysis tends to **short-lived** (resolves with resolution of infection) and because some of the hemolysis occurs intravascularly (**spleen not involved in the intravascular component of hemolysis**).

**(NB: EBV infection (mononucleosis) in young people may also be associated with this same kind of cold, IgM-mediated hemolysis.** In the case of EBV, the IgM antibody is directed against the “i” (little “I”) Ag on RBCs. Similar clinical picture of hemolysis.)

- **Which therapeutic options are appropriate for these patients?**
  - **Folate:**  regardless of the cause of **hemolysis, demand for folic acid is increased** (needed for DNA synthesis and cell turnover) to support the bone marrow’s compensatory increase in RBC production. Folate supplementation is vital to avoid folate deficiency which would compromise bone marrow production of cells. Both patients should get folate.
  - **Corticosteroids: Corticosteroids** are **immunosuppressive** medications with multiple mechanisms of action (including effects on T cell, B cell and macrophage function) **and are considered first line treatment for warm IgG-mediated AIHA.** Steroids have a multitude of serious side effects, but up to 90% of patients (with warm AIHA) will have an initial clinical response**. Steroids would be appropriate therapy for Patient 1.** **Patient 2** has a cold**, IgM-mediated hemolysis precipitated by an acute infection.** This is a **self-limited condition. Hemolysis resolves with resolution of the infection**. Steroids have not been shown to be very effective in cold agglutinin hemolysis perhaps in part because **steroids have no effect on the complement mediated intravascular component of hemolysis**. Given their toxicity and lack of efficacy, steroids **would not be indicated for patient 2.**
  - **Splenectomy** **removes the site of clearance of antibody coated RBCs**. It doesn’t impact the underlying cause. In **patients with IgG AIHA, splenectomy may be used after other therapies, such as intravenous immunoglobulin and Rituximab have failed.** Because Patient 2’s hemolysis will resolve on its own, splenectomy would not be indicated. In addition, removing the spleen would not impact the intravascular component of the complement-mediated hemolysis.
  - **Transfusion:** this would be an option in either patient if anemia was severe.

**(NB:** Patients with autoantibodies present complex issues for blood bank compatibility testing and transfusion well beyond the scope of what non-blood bank hematologists (such as me) and medical students (such as you) need to know).
